# PXR interaction with p53: a meeting of two masters

**DOI:** 10.1038/cddis.2016.122

**Published:** 2016-05-05

**Authors:** D Robbins, J Bakke, M T Cherian, J Wu, T Chen

**Affiliations:** 1 Department of Chemical Biology and Therapeutics, St. Jude Children's Research Hospital, Memphis, TN, USA

Multidrug resistance is an impending yet complex barrier to chemotherapy. The cellular response to xenobiotics, such as prescription medications, involves inducing drug-metabolizing enzymes and drug transporters that eliminate the toxicity of certain therapeutic agents or increase the risk of potential drug–drug interactions.^1^ However, given that drug metabolism and transport are necessary to maintain physiologic homeostasis, our report in *Cell Death Discovery* explores the effects of the master xenobiotic regulator, pregnane X receptor (PXR), on the master regulator of cell death, p53. Our results illustrate how a xenobiotic receptor can regulate cell death signaling through protein–protein interaction.^2^

PXR is a well-established xenobiotic nuclear receptor that has a central role in xenobiotic metabolism and disposition.^3, 4, 5^ PXR exerts its metabolic function by regulating the expression of phase I and II drug-metabolizing enzymes and drug transporters.^6^ Agonists of PXR, including structurally diverse xenobiotics, such as prescription drugs, bile acids, herbal medicines, environmental toxins, and steroids, can bind to and activate PXR.^1^ Activated PXR binds to the PXR response element within the promoter of its target gene, as a heterodimer with the retinoid X receptor to activate transcription. Therefore, xenobiotics may induce the expression of genes involved in drug metabolism or transport in a PXR-dependent manner to trigger the xenobiotic response (Figure 1a). However, emerging evidence suggests PXR is a regulator of apoptosis, with PXR activation correlating with the suppression of expression of apoptotic genes and the promotion of proliferation, drug resistance, and a malignant phenotype both *in vitro* and *in vivo*.^7, 8^

Under the normal physiologic conditions, p53 is maintained at low levels via the E3 ligase MDM2, which primes p53 for proteasomal degradation by ubiquitination.^9^ However, p53 can be activated in the presence of cellular stress that induces the DNA damage. As a transcription factor, p53 can bind to p53 response elements within the promoters of its target genes, which are known to induce the cell cycle arrest to allow for the DNA damage repair. Ultimately, if the DNA damage is irreparable, p53 can utilize a similar mechanism of transcriptional activation to induce the expression of proapoptotic genes and apoptosis (Figure 1b).^10, 11^ Our laboratory recently identified p53 as a novel protein-binding partner of PXR and dissected the role of the p53–PXR protein–protein interaction in regulating PXR activity.^12^ We demonstrated that p53 can associate with PXR and downregulate its transcriptional activity, as indicated by the downregulation of *CYP3A4* expression (Figure 1). However, although the loss-of-function p53 mutant R175H interacts with PXR, the interaction does not downregulate the transcriptional activity of PXR. We further demonstrated that the mutated p53 can reduce the suppressive effect of wild-type p53 by competitive interaction with PXR, suggesting that the protein–protein interaction is required, but not sufficient for p53 to inhibit PXR. Moreover, PXR▵174–210, a naturally occurring PXR variant with a deletion of a conserved, unique sequence in the ligand-binding domain, failed to interact with p53, suggesting that the PXR–p53 interaction is specific to wild-type PXR. We also showed that the p53-mediated suppression of PXR-mediated *CYP3A4* expression was mediated by reduced binding of PXR to the *CYP3A4* promoter.^12^

Zhou *et al.*^7^ demonstrated the antiapoptotic role of PXR in human colon cancer cells, providing a potential mechanistic connection between xenobiotic metabolism and apoptotic signaling. To understand the antiapoptotic function of PXR in regulating p53 signaling, we further explored the mechanistic link between PXR and p53. We demonstrated that PXR expression reduced p53 transactivation and the expression of its downstream target genes involved in cell cycle arrest and apoptosis.^2^ Similar to previous findings from our laboratory,^12^ PXR expression significantly decreased p53 recruitment to the promoter regions of various p53 target genes, including *CDKN1A* and *MDM2*.^2^ We also showed that the elevated PXR expression decreased doxorubicin- and nutlin-3a-mediated toxicity, and promoted malignant transformation in colon cancer cells. However, our *in vitro* models may have been biased for PXR–p53 interaction and excluded the involvement of systemic drug metabolism. When using *in vitro* or *in vivo* models in which PXR-mediated drug metabolism occurs, we must consider the effect of PXR-mediated induction of drug-metabolizing enzymes and drug transporters on the PXR–p53 protein–protein interaction, especially when p53 activation is mediated by substrates of PXR-induced drug-metabolizing enzymes. Ouyang *et al.*^13^ demonstrated that PXR expression in colon cancer cells, containing mutated p53 suppressed their proliferation and tumorigenicity. However, whereas mutated p53 might interact with PXR,^12^ whether and how PXR affects the function of mutated p53 is unknown. We made our observations in human colon cancer cells harboring wild-type p53, in which PXR expression inhibited wild-type p53, decreased expression of genes involved in cell death and cell cycle arrest, and promoted malignant transformation.^2^ Overall, these results suggest a mutually inhibitory relationship between PXR and p53. Thus, the master regulators PXR and p53 appear to have important yet opposing roles in the cellular response of tumor cells to chemotherapy.

The inhibitory effect of PXR on p53 may have broad implications for our understanding of PXR-regulated responses and the role of PXR-mediated protein–protein interactions in treating human disease. Gotoh and Negishi^14^ recently characterized the novel statin/PXR/serum/glucocorticoid regulated kinase 2 (SGK2)-mediated signaling pathway that occurs in hepatic gluconeogenesis. They reported that PXR acts as a scaffold for phosphatase 2C and SGK2 to induce dephosphorylation of SGK2, thereby promoting PXR-mediated activation of gluconeogenic genes in human liver cells and enhancing gluconeogenesis. These findings provide insight into the involvement of PXR-mediated protein–protein interactions in the increased risk of type 2 diabetes associated with statin treatment.^14^

The mutual inhibitory effect of PXR–p53 interaction is consistent with the tumor-suppressive function of p53 and the oncogenic function of PXR. p53 induces apoptosis and inhibits PXR to decrease drug metabolism and enhance drug efficacy, thereby helping to increase cancer cell death in response to chemotherapy. In contrast, PXR enhances drug metabolism to decrease drug efficacy and inhibits p53 to decrease apoptosis, thereby contributing to drug resistance. The outcome of the PXR–p53 interaction is, therefore, complex. Because PXR can be activated by many xenobiotics, whereas p53 can be activated by various cellular stressors, including xenobiotics such as DNA-damaging agents, the regulation of PXR–p53 interaction warrants further investigation.

## Figures and Tables

**Figure 1:**
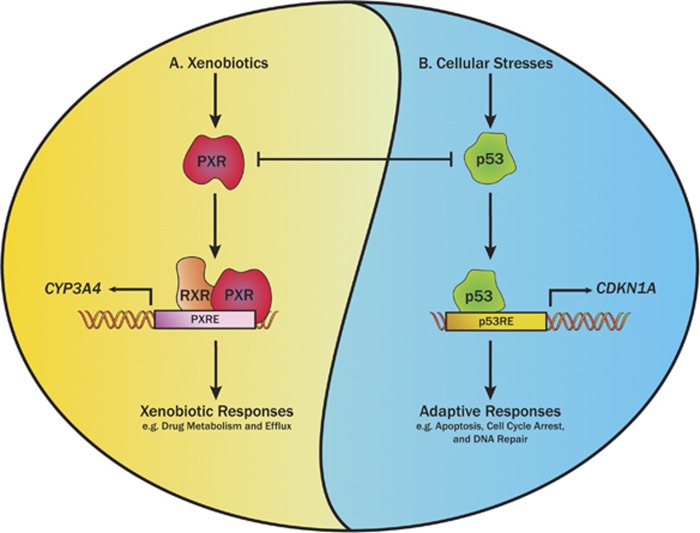
A schematic representation of the proposed PXR–p53 protein–protein interaction and its effects on the PXR-mediated xenobiotic responses and the p53 proapoptotic signaling pathway. A. Exogenous xenobiotics can act as agonists of pregnane X receptor (PXR). PXR forms a heterodimer with the retinoid X receptor (RXR), binds to the PXR response element (PXRE) within the promoters of its target genes, and activates the expression of drug-metabolizing enzymes, such as CYP3A4, to induce the xenobiotic responses. B. Cellular stresses can induce DNA damage, resulting in the activation of the tumor suppressor p53. Once activated, p53 binds to the p53 response element (p53RE) within the promoters of its target genes to induce the expression of genes involved in cell cycle arrest, such as *CDKN1A*, or proapoptotic genes that are involved in apoptosis to induce cell death. The protein–protein interaction of PXR and p53 is mutually repressive: p53 physically binds to PXR and reduces *CYP3A4* expression by decreasing the binding of PXR to the PXRE within the *CYP3A4* gene promoter, whereas PXR reduces p53-mediated transcription activity by physically binding to p53 and decreasing the binding of p53 to p53RE within p53 target genes
